# A Radial Glia Fascicle Leads Principal Neurons from the Pallial-Subpallial Boundary into the Developing Human Insula

**DOI:** 10.3389/fnana.2017.00111

**Published:** 2017-12-05

**Authors:** Emilio González-Arnay, Miriam González-Gómez, Gundela Meyer

**Affiliations:** ^1^Unit of Pathology, Department of Basic Medical Science, Faculty of Medicine, University of La Laguna, San Cristóbal de La Laguna, Spain; ^2^Unit of Anatomy, Department of Basic Medical Science, Faculty of Medicine, University of La Laguna, San Cristóbal de La Laguna, Spain; ^3^Unit of Histology, Department of Basic Medical Science, Faculty of Medicine, University of La Laguna, San Cristóbal de La Laguna, Spain

**Keywords:** cytoarchitecture, inner granular layer, pallial-subpallial boundary, lateral cortical stream, migration, radial glia

## Abstract

The human insular lobe, in the depth of the Sylvian fissure, displays three main cytoarchitectonic divisions defined by the differentiation of granular layers II and IV. These comprise a rostro-ventral agranular area, an intermediate dysgranular area, and a dorso-caudal granular area. Immunohistochemistry in human embryos and fetuses using antibodies against PCNA, Vimentin, Nestin, Tbr1, and Tb2 reveals that the insular cortex is unique in that it develops far away from the ventricular zone (VZ), with most of its principal neurons deriving from the subventricular zone (SVZ) of the pallial-subpallial boundary (PSB). In human embryos (Carnegie stage 16/17), the rostro-ventral insula is the first cortical region to develop; its Tbr1+ neurons migrate from the PSB along the lateral cortical stream. From 10 gestational weeks (GW) onward, lateral ventricle, ganglionic eminences, and PSB grow forming a C-shaped curvature. The SVZ of the PSB gives rise to a distinct radial glia fiber fascicle (RGF), which courses lateral to the putamen in the external capsule. In the RGF, four components can be established: PF, descending from the prefrontal PSB to the anterior insula; FP, descending from the fronto-parietal PSB toward the intermediate insula; PT, coursing from the PSB near the parieto-temporal junction to the posterior insula, and T, ascending from the temporal PSB and merging with components FP and PT. The RGF fans out at different dorso-ventral and rostro-caudal levels of the insula, with descending fibers predominating over ascending ones. The RGF guides migrating principal neurons toward the future agranular, dysgranular, and granular insular areas, which show an adult-like definition at 32 GW. Despite the narrow subplate, and the absence of an intermediate zone except in the caudal insula, most insular subdivisions develop into a 6-layered isocortex, possibly due to the well developed outer SVZ at the PSB, which is particularly prominent at the level of the dorso-caudal insula. The small size of the initial PSB sector may, however, determine the limited surface expansion of the insula, which is in contrast to the exuberant growth of the opercula deriving from the adjacent frontal-parietal and temporal VZ/SVZ.

## Introduction

**FIGURE 1:**
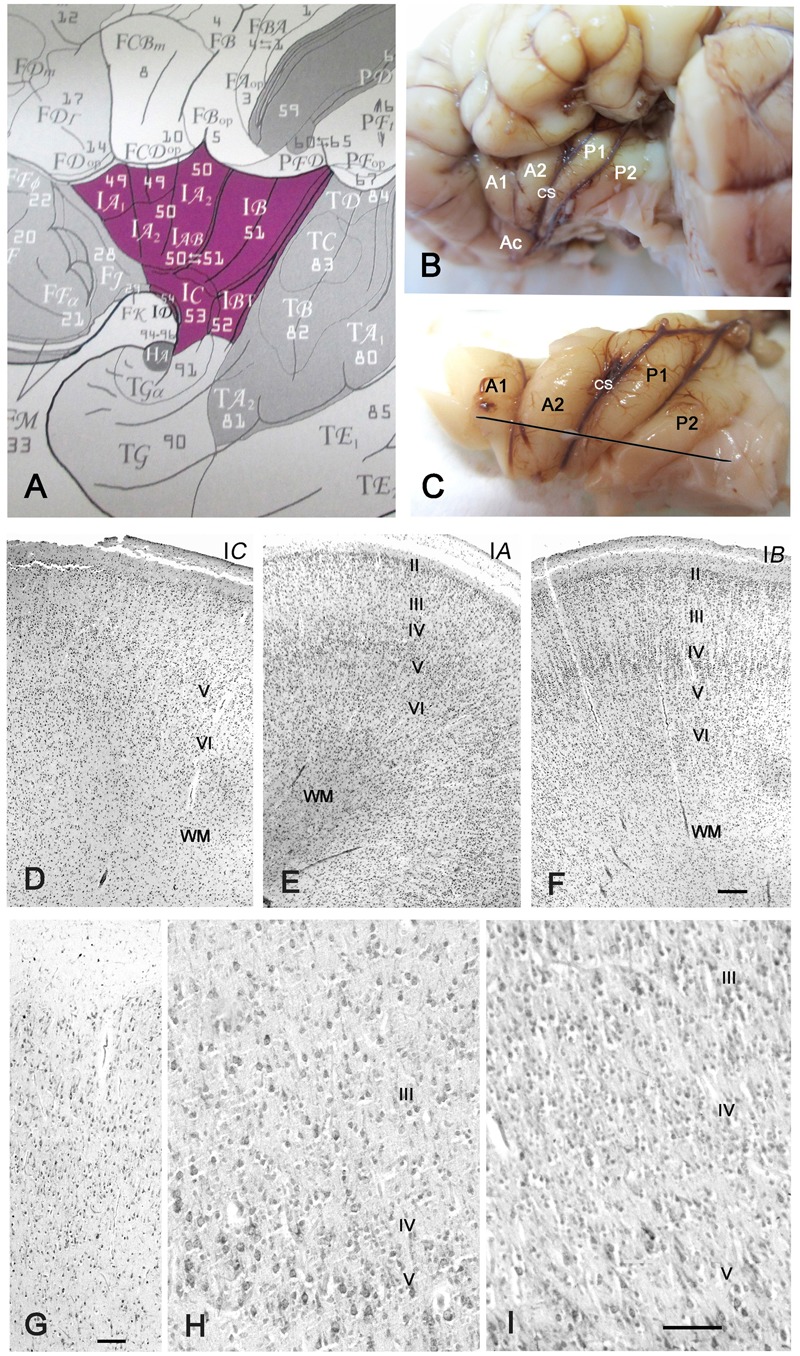
Cytoarchitecture of the perinatal human insula. **(A)** Classification of the insular areas of Von Economo and Koskinas (1925). I*A*, anterior agranular, I*B*: posterior granular, I*AB* intermediate dysgranular areas. **(B)** Left insular lobe of a newborn infant (40 GW) after removing the anterior temporal pole. A1, A2, anterior short gyri; Ac: accessory short gyrus; P1, P2, posterior long gyri; CS: central sulcus of the insula. **(C)** Dissection of the insula in **B**. The line indicates the plane of section. **(D–F)** Nissl-stained sections from the brain in **B,C**. **(D)** I*C*, the agranular transition area between the POC and the isocortical insula; **(E)** Area I*A*, representing the dysgranular area, with an irregular layer IV; **(F)** area I*B*, the granular posterior insula. Notice that layer II is still cell-dense and not yet fully mature. **(G–I)** 32 GW. **(G)** Transition between POC and isocortical insula showing ill-defined layering. **(H)** A poorly developed layer IV in the dysgranular insula; **(I)** A wide, cell-rich layer IV in the granular insula. Bars: in **F**, for **D–F**: 160 μm; in **G**: 50 μm; in **I**, for **H** and **I**: 55 μm.

The human insular lobe lies in the depth of the Sylvian fissure and is hidden by the opercula of the adjacent cortical areas. The dorsal operculum is formed successively by prefrontal (PF), frontal cortex (FC) and parietal cortex (PC), the ventral operculum by the temporal cortex (TC). The limen insulae represents its boundary with the primary olfactory cortex (POC), as well as the junction of the temporal lobe with the ventral insular cortex (Mesulam and Mufson, 1985). The circular or limiting sulcus forms the border between the opercula and the insula. The macroscopic anatomy of the insular lobe has been described in detail (Türe et al., 1999; Naidich et al., 2004; Tanriover et al., 2004). Similarly, the microscopic structure of the human (and non-human primate) insula has been the subject of numerous cytoarchitectonic studies, which distinguish a variable number of cytoarchitectonic subdivisions, ranging from the subdivision into an anterior and a posterior insula by Brodmann (1909) to the 31 areas identified by Rose (1928) [for review, see Nieuwenhuys (2012)]. We followed the widely accepted organization of the insula into concentric belts of increasing granularity (degree of prominence of the granular layers IV and II) around the POC (Mesulam and Mufson, 1985), established in the monkey but also valid in human. This concept is similar to the areas established by Von Economo and Koskinas (1925; **Figure 1A**). The insula is continuous with the allocortical POC through a periallocortical agranular field I*A*, which predominates in the anterior insula, an isocortical granular field I*B*, that occupies the caudal insula, and a large intermediate dysgranular region termed I*AB*. More recent cytoarchitectonic studies (Morel et al., 2013) confirmed the tripartite classification of Mesulam and Mufson (1985).

The connectivity of the insula with thalamus, other cortical areas, ventral striatum, hypothalamus and amygdala, has also been studied extensively (reviewed by Augustine, 1996, and Nieuwenhuys, 2012). Through the thalamus, gustatory, vestibular, visceroceptive, nociceptive, and thermoceptive information reach different parts of the insula, where they converge with information from limbic centers and the brain stem. Functional neuroimaging revealed that the insula forms part of distributed neuronal networks involved in complex cognitive functions. (For reviews and meta-analyses, see Kurth et al., 2010; Cauda et al., 2011; Deen et al., 2011; Fan et al., 2011; Nieuwenhuys, 2012). Craig (2009, 2010, 2011) proposed a concept of insular function where salient information is conveyed stepwise from posterior to anterior insular levels, converging at each step with polymodal information and cortico-cortical afferents, and with the anterior insula representing the neural substrate of awareness. Electrical disruption of the left anterior-dorsal insula/claustrum selectively impaired conscious awareness (Koubeissi et al., 2014). A brain network between the left rostral dorsolateral pontine tegmentum and the left anterior insula and anterior cingulate cortex is involved in wakefulness and awareness; brain stem lesions disconnecting this network lead to coma or disorders of consciousness (Fischer et al., 2016). Interestingly, both anterior insula and anterior cingulate cortex are populated by the von Economo neurons (VEN), spindle shaped projection neurons in layer V (Allman et al., 2011). Anterior insula and anterior cingulate cortex have a close functional relationship and may belong to a neural system engaged in multiple cognitive, affective, and behavioral contexts (Medford and Critchley, 2010).

Anatomical and functional alterations of the insula have been related to important human pathologies. According to Bonthius et al. (2005), the insular subdivisions are differently affected by neurofibrillary tangles in Alzheimer’s disease; the agranular region is more affected than the dysgranular region, whereas the granular insula is the less affected. Schizophrenic patients have a volume reduction of the insular cortex, with larger reductions of the anterior insula (Shepherd et al., 2012), which is particularly severe in patients with childhood-onset schizophrenia (Moran et al., 2014). Atypical patterns of insula activation, in particular hypoactivity of the right anterior insula, and dysfunctional insular connectivity was also observed in autism spectrum disorder (ASD) (Di Martino et al., 2009; Uddin and Menon, 2009; Odriozola et al., 2016).

In view of the impressive amount of data on the structure and function of the adult human insula, the almost absence of developmental studies is surprising. In the early literature, the insula was considered the first cortex to differentiate (Streeter, 1912; Kodam, 1926), which is in line with a more recent report showing that sulcation, gyration, and vascularization of the human cortex start in the insular region (Afif et al., 2007). We describe here the development of the human insula from early embryonic stages to term by using immunohistochemistry for radial glia markers vimentin (Ulfig et al., 1999; Zecevic, 2004) and nestin (Zecevic et al., 2005), PCNA and Tbr2 (Englund et al., 2005) for cell proliferation and pallial progenitors, respectively, and Tbr1 as a marker of pallial neurons (Hevner et al., 2003).

Our main questions were: Where do the progenitor cells of the insula come from? How can migrating neurons reach the insula, which is so far away from the proliferating zones of the cortex? Which mechanisms can possibly explain the distinct lamination patterns of the insular subdivisions? Our analysis of the radial glia architecture in the developing telencephalon suggests that the principal neurons of the insula derive from the pallial-subpallial boundary (PSB) and migrate along a radial glia fascicle (RGF) connecting the PSB with the insula. The RGF follows the curvature of the PSB, and serves as a migration substrate for migratory neurons from the PF, frontal, parietal, and temporal PSB into the insula, with descending radial glia fibers partially merging with ascending ones. The diversity of radial glia fiber origins and trajectories might underlie the cytoarchitectonic diversity of the human insula.

## Materials and Methods

The fetal human brains, between 9 and 25 gestational weeks (GW): 9 GW (2), 10 GW (3), 11GW (4), 12 GW (3), 13 GW (2), 14 GW (2), 15 GW (3), 16 GW (4), 17 GW (2), 18 GW (1), 19 GW(1), 20 GW (2), 21 GW(6), 22 GW (3), 23GW (2), 24 (1), and 25 GW (1) were from our collection used in previous studies (e.g., Meyer et al., 2000; González-Gómez and Meyer, 2014; Meyer and González-Gómez, 2017). The embryonic cases, 5.5–8.5 GW, are the same described in Meyer et al. (2000). They were obtained after legal abortions following national guidelines in Spain, under the supervision of the Ethical Committee of the University of La Laguna, in accordance with the Declaration of Helsinki, 1964. Written informed consent was obtained from the parents for the use of embryonic and fetal brains. The embryos were staged according to Carnegie stages (CS) defined by O’Rahilly and Müller (1994). The perinatal brains, 32 GW (1 case) and 40 GW (3 cases) were from children without known neurological pathologies that died during or shortly after birth. The embryonic and fetal brains were fixed in Bouin or Carnoy, embedded in paraffin, and cut in a coronal or, in four cases, in a horizontal plane into 10 μ-thick serial sections.

Due to their large size, the perinatal brains were cut into blocks, most of which were cut coronally. In the 32 GW case and one 40 GW case, the insula was dissected out (**Figures 1B,C**) and cut in a plane considered almost perpendicular to the main axis of most insular gyri (**Figure 1C**), as recommended by Von Economo and Koskinas (1925) for an optimal visualization of cytoarchitecture.

### Immunohistochemistry

Sections were deparaffinized, hydrated, and boiled in 10 mM citrate buffer (pH 6) for 20 min for antigen retrieval, rinsed in Tris-buffered saline (TBS, pH 7.6, 0.05 M), and incubated in the primary antibodies overnight in a humid chamber. After rinsing, they were incubated in the corresponding biotinylated secondary antibodies (rabbit anti-mouse IgG or goat anti-rabbit IgG; Dako, Glostrup, Denmark), diluted at 1:200 in TBS, followed by incubation with avidin-biotin complex (ABC, DAKO) in TBS. Bound peroxidase was revealed using 0.04% 3,3-diaminobenzidine (Sigma, United States), 0.05% ammonium nickel (II) sulfate, and 0.03% hydrogen peroxide in TBS, pH 7.6. Sections were dehydrated, cleared, and coverslipped using Eukitt (O. Kindler, Freiburg, Germany). Negative controls omitted the primary antibodies.

The following primary antibodies were used: Mouse monoclonal anti-reelin antibody 142 [IgG1, 1:500, (gift of A. Goffinet), 1/500; Rabbit polyclonal anti-Calretinin, Swant, 7699/4, 1/3000; Mouse monoclonal antibody anti-PCNA, Thermo Scientific, Ab-1 (clone PC10) 1/1000; Rabbit polyclonal anti-Tbr1, Abcam, ab31940, 1/300; synthetic peptide within human Vimentin aa 400 to the C-terminus (acetyl), 1/200, Abcam]; Rabbit polyclonal anti-nestin, Abcam, ab 93666, 1/100; Rabbit polyclonal anti-MAP2, Sigma, HPA 012828, 1/100; Rabbit polyclonal anti-Eomes (Tbr2) Sigma, HPA028896, 1/100.

### Sequential Two Color Immunostaining

Antigens were immunolabeled sequentially by using primary antibodies (Tbr1 and CR; CR and PCNA) generated in rabbit. The first antibody was developed using DAB/nickel as chromogen. Thereafter, sections were rinsed in TBS and incubated overnight with the second antibody. After incubation with the biotinylated secondary antibodies and ABC as described above, sections were developed by using DAB alone as chromogen. Sections were dehydrated, cleared in xylene, and cover-slipped with Eukitt (Freiburg, Germany). Photographs were taken with a Zeiss Axio microscope equipped with an AxioCam MRc5 digital camera and AxioVision LE 4.6 software. Images were processed using Adobe Photoshop CS2 for adjustment of brightness and contrast.

## Results

### Gyration and Cytoarchitecture of the Perinatal Insula

The insula of perinatal (32–40 GW) brains displayed an adult-like gyration pattern (**Figures 1B,C**), although the case in **Figures 1B,C**, 40 GW) presented only two short anterior gyri with one accessory anterior gyrus, and two long posterior gyri on each side. Another 40 GW brain and the 32 GW case showed the more common configuration of three short anterior and two long posterior gyri. As in the adult (Mesulam and Mufson, 1985), three main modalities of insular cytoarchitecture were recognizable at term, following a gradient from rostro-ventral to caudo-dorsal, independently of the sulcal pattern: The antero-basal sector of the insula near the limen (Field I*C* of Von Economo and Koskinas, 1925; **Figure 1A**) was agranular, showing a prominent layer V but absence of the inner granular layer IV (**Figure 1D**). At levels rostral to the limen, the insular cortex was continuous with the POC via a small transition area where the neurons lacked any recognizable lamination, with superficial medium-sized pyramidal cells and deeper smaller pyramidal and non-pyramidal cells distributed apparently at random. The anterior gyri had a variable prominence of layer IV and were thus considered dysgranular (**Figure 1E**), whereas in the posterior and dorsal insula layer IV was wider, more cell-dense, and radially organized (**Figure 1F**), features characteristic of a granular isocortex. Nonetheless, the width of layer IV was variable, even along the same gyrus. The local heterogeneities of layer IV may reflect the immaturity of the perinatal brain, but may also be due to laminar distortions when a gyrus changes orientation or undergoes additional folding.

At 32 GW, the insula showed the same basic folding and lamination pattern as at 40 GW, even though neurons appeared slightly less mature, with a higher cell density than at term. Transitional (**Figure 1G**), dysgranular (**Figure 1H**) and granular (**Figure 1I**) regions were clearly established. At both 32 and 40 GW, the outer granular layer II (**Figures 1E,F**) was more cell-dense than in the adult, due to the inside-out migration gradient of the cortex, according to which layer II is the last layer to develop (Angevine and Sidman, 1961; Rakic, 1974).

We conclude that the insula acquires an adult-like gyration and architectonic pattern during the last trimester of gestation. We did not detect the VEN (Allman et al., 2011), possibly because our material did not include the fronto-insular transition area where they are more numerous, and because they mature at later stages (Allman et al., 2005).

### Early Stages of Insular Development Prior to the Appearance of the Sylvian Fissure

#### The Lateral Cortical Stream

Since classical studies (Streeter, 1912; Kodam, 1926) proposed that the insula is the first cortical area to appear, we examined the early stages of telencephalic development from 5.5 GW onward. While in the later stages the insular lobe is defined by the presence of the Sylvian fissure, in the embryonic and early fetal stages the future insular territory was more difficult to identify, recognizable only as the transition area between the POC and the prospective isocortex, and by its position external to the developing putamen.

**FIGURE 2:**
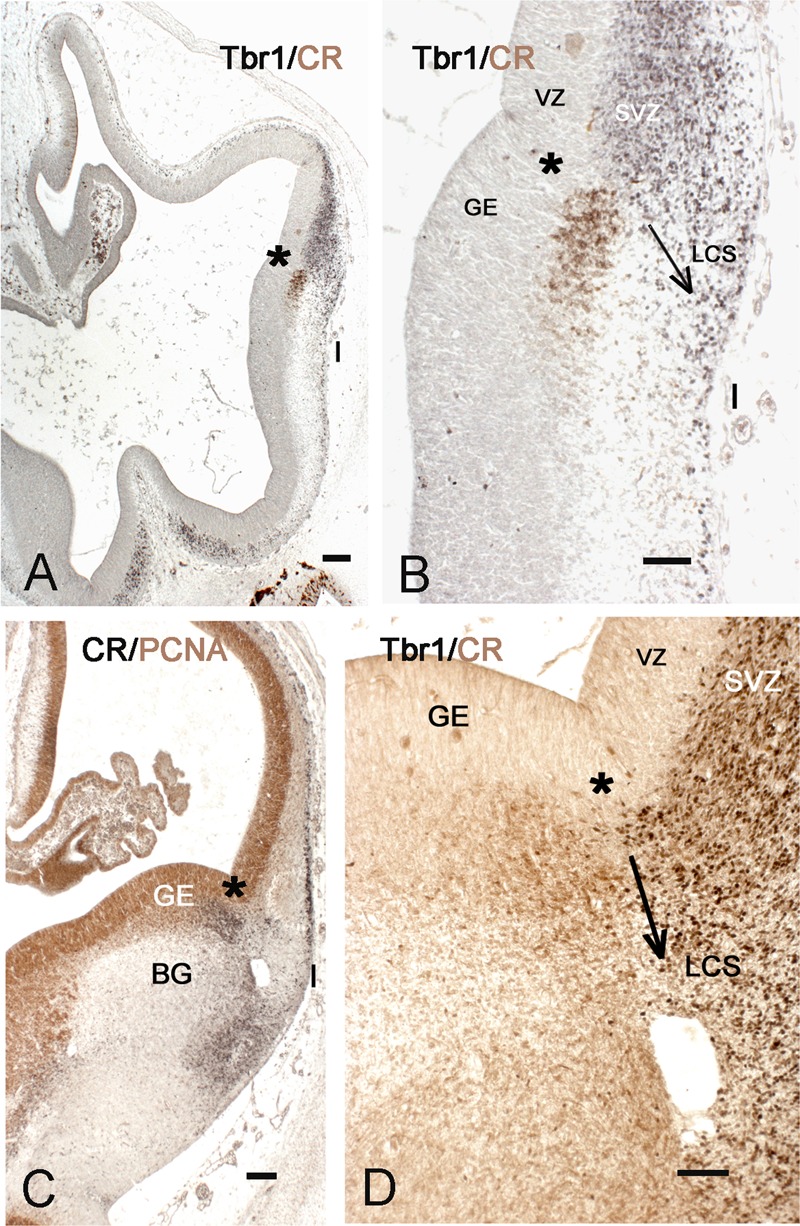
The early appearance of the insula and the lateral cortical stream (LCS). **(A,B)** Telencephalon of a 5.5 GW embryo, double-stained with Tbr1 (black) and calretinin (brown). Tbr1+ pallial cells descend from the PSB (asterisk) to the future POC and insula (I), representing the pallial part of the LCS. The cortex is still in the early preplate stage. Calretinin+ cells in the subventricular zone (SVZ) of the lateral ganglionic eminence (LGE) form the subpallial component of the LCS. **(C,D)** PSB and LCS at Carnegie stage (CS) 19, double-stained for PCNA (brown) and calretinin (black) in **C**, and for Tbr1 (black) and calretinin (brown) in **D**. The calretinin+ cells at the PSB and in the POC are mainly post-mitotic; both the pallial, Tbr1+, and the subpallial, calretinin+ components of the LCS have largely increased in size, along with the growth of the SVZ at the PSB. The arrows in **B,D** indicate the proposed direction of the LCS. BG, basal ganglia; GE, ganglionic eminences. Bars: in **A,C**: 100 μm; in **B,D**: 50 μm.

A key structure at this stage is the lateral cortical stream (LCS) (Bayer and Altman, 1991), a migration pathway that originates at the PSB and leads toward the olfactory forebrain, running in a position lateral to the putamen. The LCS appeared as early as CS 16/17 (5.5 GW) (**Figures 2A,B**) at the PSB, situated slightly medial to the cortico-striatal sulcus, and represented the first Tbr1+ migration stream of the developing pallium, while the cortical anlage was still in the preplate stage, and the ganglionic eminences (GE) visible only as small elevations in the lateral ventricle. The pallial LCS migration was more massive than the subpallial one, which appeared as a small patch of calretinin+ cells in the subventricular zone (SVZ) of the lateral ganglionic eminence (LGE). Both components of the LCS were segregated and not overlapping. In this initial stage, the Tbr1+ stream extended ventrally toward the pial surface of the prospective insula and POC, whereas the calretinin+ stream had not yet left the SVZ of the LGE.

**FIGURE 3:**
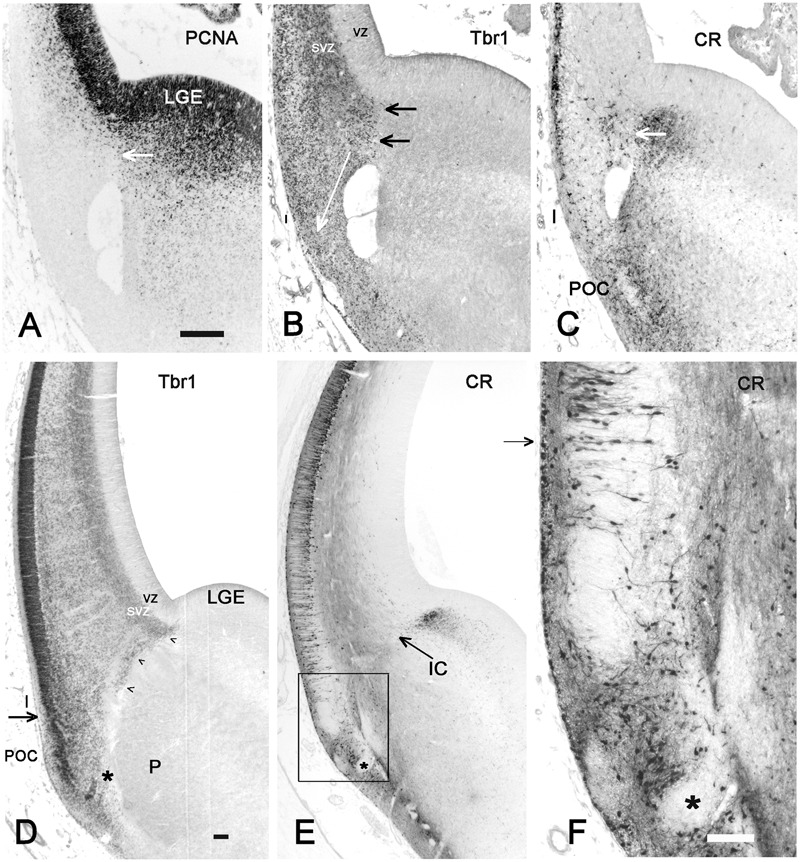
Insula and POC derive from the PSB. **(A–C)**: 7 GW, **(D–F)**: 9 GW. **(A)** PCNA shows dividing cells in the new appeared SVZ of the lateral cortex and in the putamen, while LCS and Insula do not contain mitotic cells. The white arrow points to the PSB. The hole in **A,C,D** is probably not an artifact since it is present in almost all brains of this age group; it may represent an early blood vessel that precedes the appearance of the IC. **(B)** Tbr1, and **(C)** calretinin are expressed on the pallial and subpallial sides, respectively, of the PSB, indicated by horizontal arrows. The white arrow in **B** shows the proposed direction of the LCS into Insula and POC. The calretinin+ cells in the lateral cortex represent the first pioneer cells of the advanced preplate and are probably unrelated to the PSB. **(D)** Tbr1, **(E)** calretinin and **F** (the inset in **E**) show the further differentiation of the PSB derivatives, and the complex cell arrangement at the POC-insula transition (arrows in **D** and **F**). We suggest that the less compact arrangement of the Tbr1+ cortical plate (CP), compared to more dorsal levels, is the anlage of the rostro-ventral insula **E** and **F** show the intermixture of pallial, Tbr1+ cells and non-pallial, Tbr1-negative cells (asterisks points to the same Tbr1+, calretinin-negative in **D–F**), in the POC/endopiriform complex. In **F**, pyramidal-like deep pioneer cells may indicate the ventral boundary of the insula. Bars: **A**, for **A–C**: 100 μm; **D,E**: 100 μm; **F**: 300 μm.

The further development of the LCS is illustrated in **Figures 2C,D** and **3A–C**. At CS 18/19 (6.5 GW), the cells forming the LCS had increased in number, concurrent with a generalized growth of the SVZ of GE and cortex anlage. The calretinin+ stream now extended ventrally and formed cell aggregates in the developing POC/endopiriform complex (**Figure 3C**). The more lateral Tbr1+ stream reached the same ventral level, but occupied the entire SVZ and ventral cortical territories (in early human corticogenesis, Tbr1 marks both SVZ progenitor cells and postmitotic migratory neurons). Concurrently, the first representatives of the calretinin+ pioneer plate (Meyer et al., 2000) had appeared in the lateral cortex (**Figure 2C**), and became more evident at CS 20 (7 GW) (**Figure 3C**). In most embryonic brains from this period, a conspicuous hole may mark the site of the future internal capsule (IC) (**Figures 3A–C**) (see also plates 190 A and B in Bayer and Altman, 2007). Mitotic figures were numerous in the striatal anlage, but absent from the LCS, which was thus a non-proliferating structure (**Figure 3A**). In the embryonic stages, the RGF [see below section Migration from the PSB to the Insula along the Radial Glia Fascicle (RGF)] had not yet formed; after its appearance around 11 GW neurons migrating into the insula used the RGF as a migration substrate, and the LCS might thus be considered as its forerunner.

At 8–10 GW, a highly complex neuronal configuration characterized the insula and adjacent POC/endopiriform area, determined following Bayer and Altman (2007). In the POC, aggregates of Tbr1+ cells were intermixed with CR+/Tbr1- neurons forming complex nuclear structures (**Figures 3D–F**). We considered the loosening of the compact Tbr1+ lateral cortical plate (CP), together with a narrowing of the marginal zone compared to the POC, as landmarks defining the territory of the insular cortex. Calretinin marked neurons with a pyramidal shape, which corresponded to the deep pioneer cells representing the presubplate (Meyer et al., 2000), in what we propose as the ventralmost extension of the prospective insula (**Figure 3F**). They were separated from the superficial pioneer neurons by calretinin-negative CP neurons. Regarding the distribution of CR+ deep pioneer neurons, the transition between insula and POC appeared as a gradual one. Importantly, at this early age of 8 GW, the anteroventral insula had already a laminated organization, indicating that its deep layers were already formed as derivatives of the PSB via the LCS.

#### The Developing Internal Capsule Crosses the PSB and Delimits the Rostro-Caudal Extent of the Insula

**FIGURE 4:**
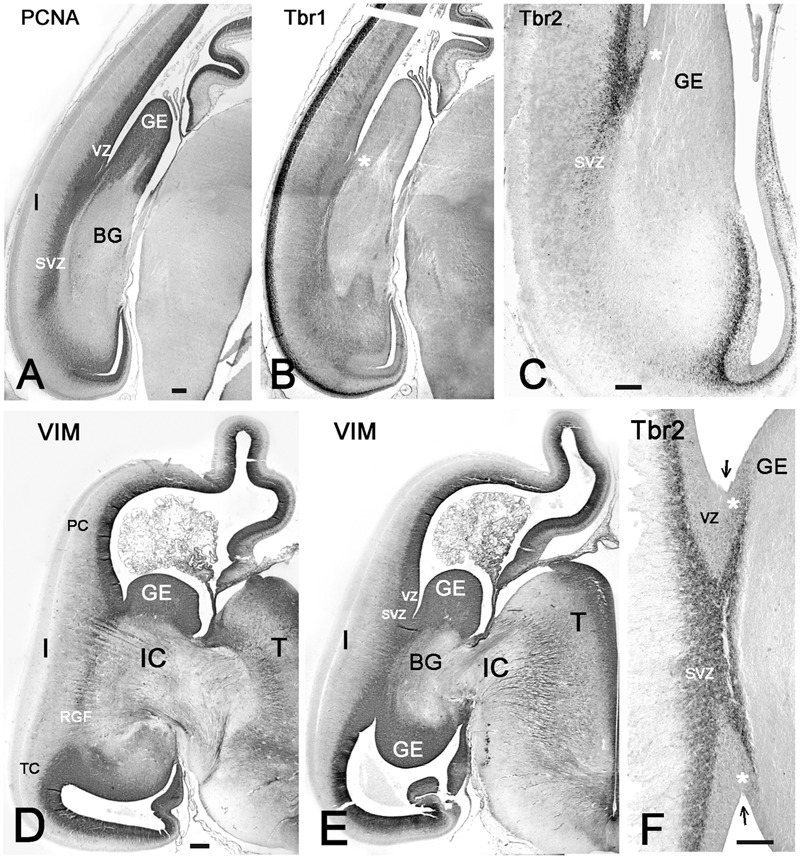
The PSB and the growth of the internal capsule (IC). **(A–C)** Coronal sections through a caudal telencephalic level at 9 GW, and in **D–F**, through two different levels at 10 GW, show the growing curvature of the lateral ventricle and the opening of the temporal horn at 10 GW. The IC enters the cortex crossing the PSB following a rostral to caudal sequence. While the IC has crossed the PSB at rostral levels (**Figure 3E**), it has not yet appeared at caudal levels, where PCNA **(A)** and Tbr2 **(C)** show mitotic cells at the PSB (indicated by white asterisks), whereas Tbr1 marks the pallial territory **(B)**. **(D)** At 10 GW, the SVZ of the fronto-parietal PSB and the temporal PSB are connected by a vimentin+ radial glia fascicle (RGF), which is traversed by the IC and delimits the insula. At this time point, the IC has not yet reached the caudal PSB **(E)**, where the SVZ is particularly wide. In **F** (same level as **E**), the PSB extends medial to the cortico-striatal sulcus (arrows). GE, ganglionic eminence; I, insula; IC, internal capsule; PC, parietal cortex; SVZ, subventricular zone; T, thalamus; TC, temporal cortex; VZ, ventricular zone. Bars: **A–F**: 100 μm.

The IC is an important landmark in the early fetal brain; at 8 GW it was recognizable at the PSB at the level of the prospective frontal cortex (FC) as a CR-negative fiber bundle (**Figure 3E**), but not at intermediate and caudal levels, where the IC had not yet approached the PSB. At 8 GW, the caudal PSB, prior to the crossing of the IC, consisted of a proliferating SVZ positive for PCNA, Tbr1, and Tbr2 (**Figures 4A–C**). Around 9/10 GW, the IC also crossed a more caudal, midinsular level of the PSB (**Figure 4D**), while its posterior limb had just entered the GE but still not reached the PSB near the parieto-temporal (PT) junction (**Figure 4E**). Concurrently, the lateral ventricle adopted a C-shaped curvature, growing in both a rostral (frontal lobe) and ventral (temporal lobe) direction. As a consequence of the ventricular curvature, in coronal sections the ventral half of the telencephalon appeared almost like a mirror image of the dorsal half (**Figures 4D–F**). The PSB followed the curvature of the lateral ventricle; its posterior limit with the caudal part of the GE was recognizable by its SVZ, which was positive for Tbr2 and vimentin (**Figures 4E,F**). At 10 GW, the SVZ of the caudal PSB prior to the crossing of the IC was particularly broad, (**Figure 4E**) and was even wider than the SVZ of its neighboring parietal and temporal areas.

**FIGURE 5:**
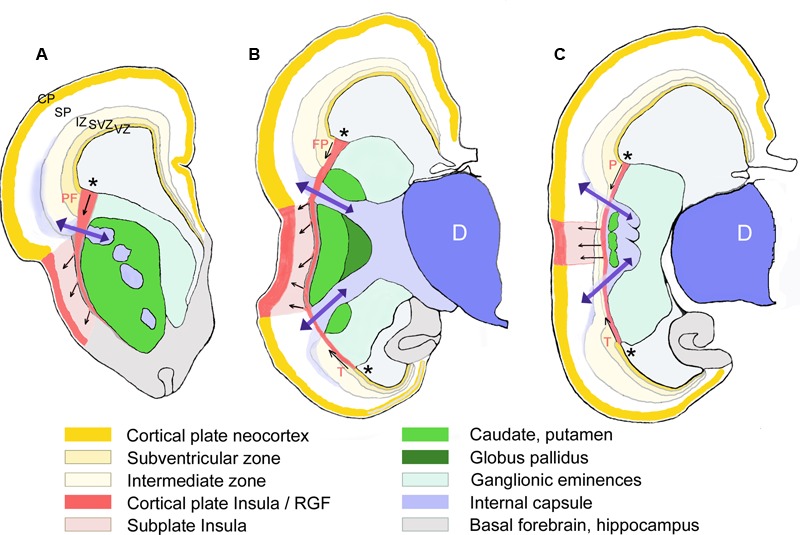
Schematic representation of the RGF leading from the PSB into the insula. Drawn from Nissl-stained sections of a 16 GW-old fetus at three different rostral to caudal levels **(A–C)**. The proposed direction of the prefrontal (PF), frontoparietal (FP), (parietal in **C**), and temporal (T) components of the RGF from the PSB (asterisks) is indicated by arrows. Only those structures mentioned in the text are represented. D, diencephalon; SP, cortical subplate (in white).

The sylvian fossa appeared at 11–12 GW, before the formation of the circular sulcus and the opercula. The insula was medially delimited by putamen and external capsule (EC), and separated from the proliferating ventricular zone (VZ) and SVZ by the growing basal ganglia (BG) and the IC, so that migrating excitatory neurons could reach the insula only indirectly via radial glia fibers crossing the IC and circumventing the putamen. To identify the insular cortex before the formation of a distinctive Sylvian fissure, we had to rely on its topographical relationships with deep structures and fiber tracts, which remain constant during development and persist into adulthood. Rostrally, the insula began at the intersection of EC and IC at the level of the PF cortex (**Figure 5A**), while caudally it was delimited by the posterior limb of the IC and the PT junction (**Figure 5C**). At this time point, the insular cortex had no distinctive features that would allow a cytoarchitectonic definition.

### Migration from the PSB to the Insula along the Radial Glia Fascicle (RGF)

**FIGURE 6:**
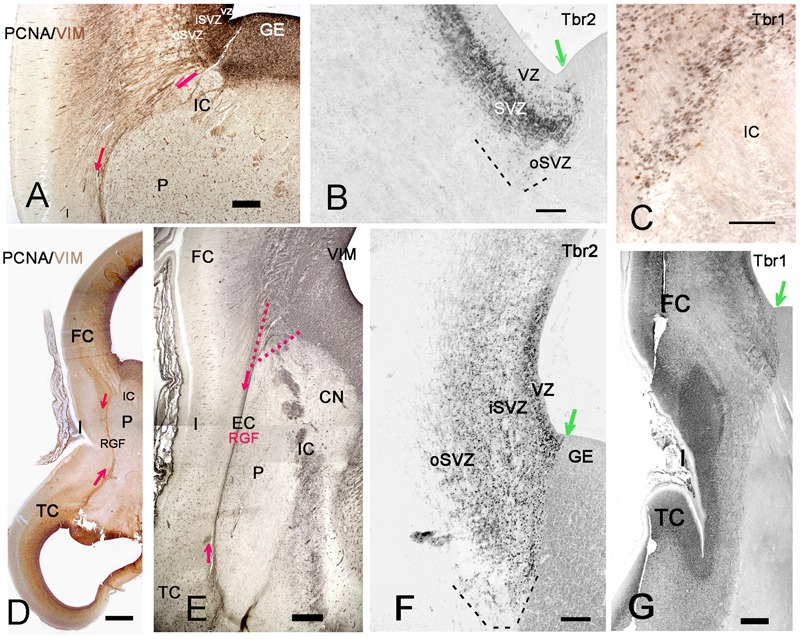
The RGF on its route to the insula. **(A,D,E)** Development of the RGF, which is co-extensive with the external capsule (EC). **(A)** At 11GW, the RGF arises from the PSB, crosses the IC and courses lateral to the putamen (P) (red arrows). PCNA (black) is expressed in the ventricular zone (VZ) and SVZ, but not in the RGF (brown). **(B)** At 11 GW, the Tbr2+ SVZ at the PSB (green arrow) extends into the IC. The dashed line indicates the outer SVZ (oSVZ) at the PSB. **(C)** Tbr1+ neurons (black) course along the RGF into the insula. Calretinin+ neurons (yellow) do not form part of this migration. **(D)** At 12 GW, the sylvian fossa indicates the position of the insula. Two-color staining (PCNA in black, vimentin in brown) shows that the RGF is not a proliferating zone. **(E)** At 15 GW, the origin of the RGF can be traced back to the intersection of IC and EC (red dotted lines), while the oSVZ has increased in width. **(F)** At 15 GW, an inner (i) and outer (o) SVZ is particularly wide at the PSB (green arrow). **G**: 14 GW, Tbr1. At this age, of incipient opercularization, Tbr1 is still expressed by cells in all cortical compartments, and clearly visualizes the separation of pallial and subpallial regions. FC, frontal cortex, TC, temporal cortex. Bars: In **A**: 270 μm; in **B**: 160 μm; in **C**: 50 μm; in **D**: 700 μm; in **E**: 350 μm; in **F**: 150 μm; in **G**: 450 μm.

We tried to identify the possible proliferative sources and their migratory routes into the insula through analysis of radial glia architecture. At 11–12 GW, basal or outer radial glia (oRG) appeared in the SVZ at the PSB, which now occupied a PCNA+ and Tbr2+ wedge-shaped area at the intersection of IC and EC. This SVZ was wider than the SVZ in the adjacent cortex (**Figures 6A,B**). Radial glia processes originating in the SVZ at the PSB were positive for vimentin and nestin, and assembled in a distinct RGF coursing along the prospective EC. Following the curvature of the PSB, the RGF originating from its SVZ formed a continuous band of radial glia fibers. According to their origin, four subdivisions of the RGF could be established, which remained constant during the first half of gestation: PF, fronto-parietal (FP), PT, and T, temporal radial glia fibers, which took different directions to reach their destination in the insula. Descending RGF component FP merged with ascending RGF component T (**Figures 5B, 6D,E**), whereas component PF descended without an ascending counterpart (**Figure 5A**). Component PT at the caudal end of the BG (putamen islands interspersed between the posterior limb of the IC) (**Figure 5C**) coursed straight to the caudal insula, where also an intermediate zone was recognizable. Along its course, the RGF fanned out and entered the insula at different dorso-ventral levels, maintaining an initially parallel orientation up to the point where descending and ascending components met (**Figure 5B**).

**FIGURE 7:**
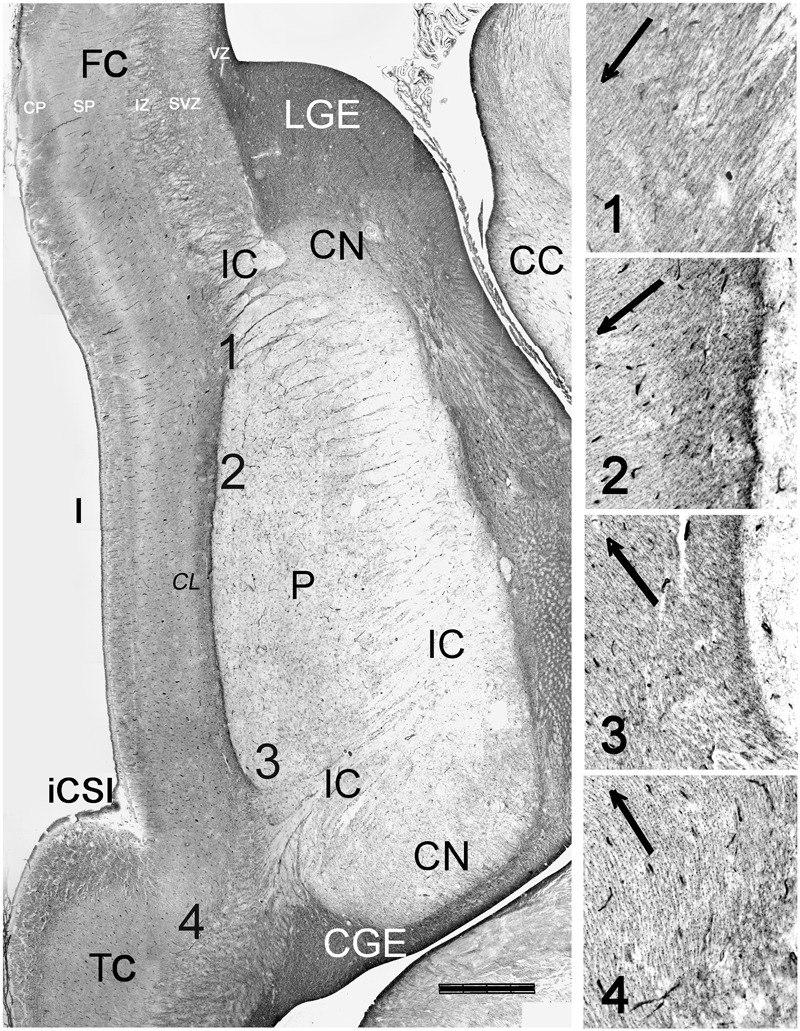
Orientation of radial glia fibers in the RGF. Reconstruction of a horizontal section at 21 GW, immunostained for vimentin. The numbers 1–4 indicate the levels represented at higher magnification, showing the dominant orientation of radial glia fibers fanning out from the RGF. Descending radial glia fibers from the fronto-parietal PSB dominate over ascending fibers from the temporal PSB. In level 4, near the temporal operculum, radial glia fibers appear to lead into the supratemporal plane rather than into the insula. The claustrum (CL) appears as a pale zone lateral to the RGF/EC. CC, corpus callosum, CGE, caudal ganglionic eminence; CN, caudate nucleus; FC, frontal cortex; CSI, circular sulcus of the insula; LGE, lateral ganglionic eminence, TC, temporal cortex. Bar: 1400 μm.

At 13/14 GW, migrating neurons, still positive for Tbr1, followed the direction of the RGF into the insula, populating its subplate and CP, clearly delimiting cortical and subcortical territories (**Figures 6C,G**). After this age, migrating neurons were Tbr1-negative. The wedge-shaped SVZ, marked with vimentin (**Figures 6D–F**), nestin, PCNA (**Figures 6A,D**), and Tbr2 (**Figures 6B,F**), progressively increased in width, partially entering the IC, and expanded in parallel with the proliferation of the outer SVZ (oSVZ) in the adjacent opercula. We reconstructed the course and orientation of the RGF components into the insula in a horizontal section at 21 GW (**Figure 7**). As expected, the RGF arose from both the dorsal and ventral oSVZ at the PSB, although the dorsal RGF predominated. The orientation of the fibers leaving the fascicle and entering the insular subplate is shown for different levels (**Figure 7**, from 1–4). At levels 1 and 2, fibers emerging from the RGF component FP took a descending course, while at level 3 (component T) they ascended. Level 4 shows radial glia fibers in the intermediate zone of the temporal operculum, which seemed to bend and course toward subplate and CP of the superior TC rather than into the insula. The RGF was compressed in the EC and contained also vimentin+ cells. However, PCNA+ mitoses were rare, and we did not observe Tbr2+ progenitor cells in this location; this indicates that the territory of the RGF was not an extension of the proliferative oSVZ.

Importantly, the PF PSB (RGF component PF) extended farther rostrally than the temporal one (RGF component T) (**Figure 5A**); in consequence, the most anterior region of the insula received radial glia fibers only from the PF PSB. The middle and posterior insular regions, in turn, received radial fiber-mediated migrations from the dorsal (FP) and ventral (temporal) PSB (**Figure 5B**). Even more caudally, the posterior end of the insula was close to the PT junction, and was also populated by large numbers of oRG cells and processes. At this level, an intermediate zone was recognizable as a fiber-rich layer continuous with the intermediate zone of the adjacent cortices (**Figure 5C**).

The distinct subregions of the insular lobe were thus connected more or less abundantly via the RGF with the proliferative SVZ along the PSB. The differential availability of progenitor cells may be the basis for the differential layering of the subareas of the insular lobe. It is remarkable that the insular lobe, despite its rather distant relationship with the proliferating oSVZ, and the limited progenitor pool at the PSB, is able to fold once migration is finished (**Figure 1B**).

### Fronto-Parietal and Temporal PSB at Midgestation

**FIGURE 8:**
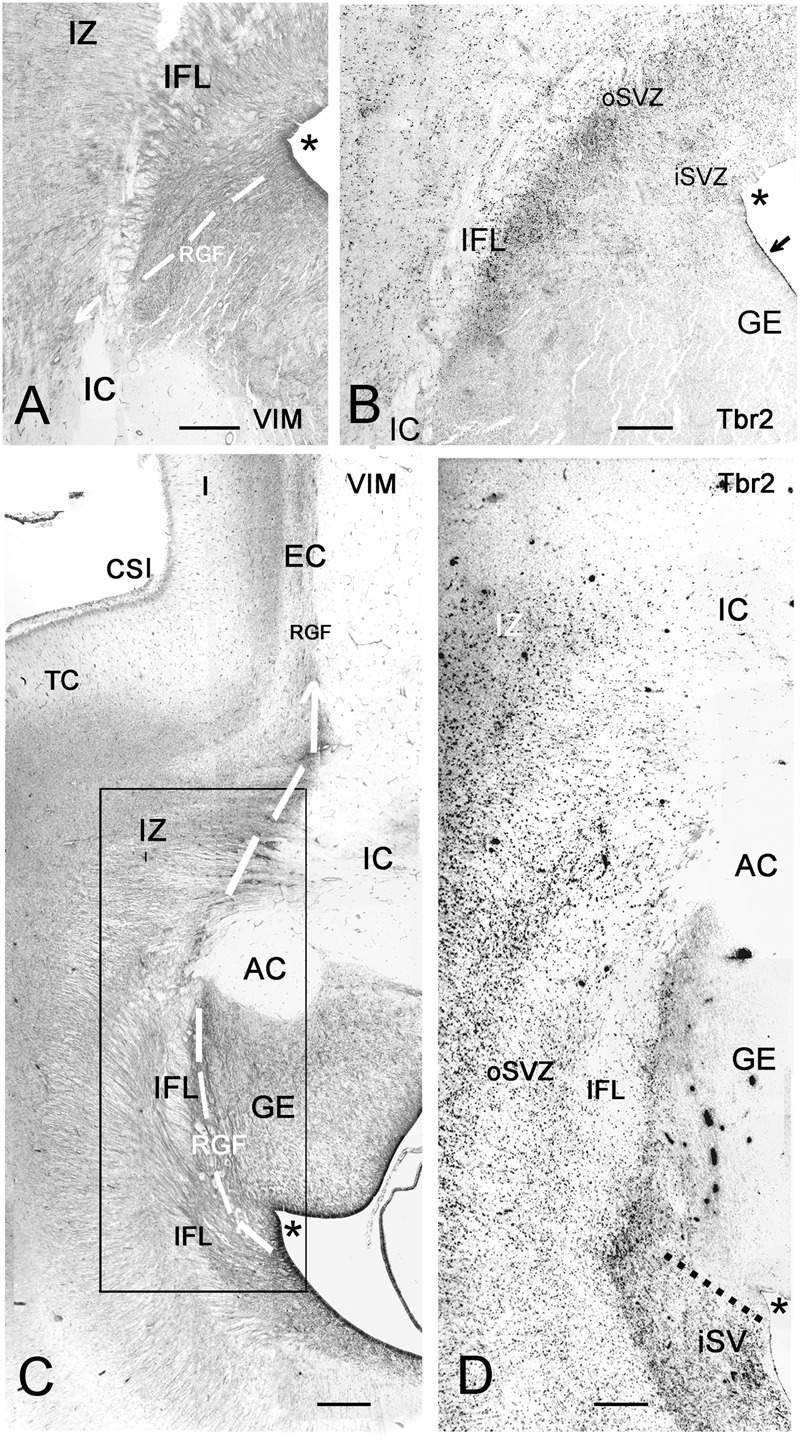
Photographic reconstructions of the PSB in coronal sections at 21 GW. Vimentin **(A,C)** shows the distribution and orientation of radial glia cells and fibers in the frontal **(A)** and temporal **(C)** periventricular zones and the RGF route into the Insula (white arrows). Tbr2 **(B,D)** marks the PSB (arrow in **B**), which at this age is less well defined than at earlier fetal stages, and in the temporal lobe does not extend medially beyond the striato-cortical sulcus (asterisks in all panels; in **D**, the PSB is indicated by a dotted line). Tbr2+ and vimentin+ progenitor cells cross the periventricular fiber layers (Inner fibrous layer, IFL) and extend far into the oSVZ and even the intermediate zone (IZ). Note the complexity of fiber tracts in the temporal lobe, due to the presence of the anterior commissure (AC). The large black dots in the inner (i) SVZ in **D** are stained blood vessels. Bars: in **A**: 240 μm, in **B**: 140 μm, in **C**: 250 μm, in **D**: 190 μm.

Vimentin, a marker of radial glia, does not define the PSB. To determine the origin of the RGF at the PSB at midgestation (21GW), we compared adjacent sections stained for vimentin and Tbr2 (**Figure 8**). When reconstructing the photomosaics for both markers from high magnification microphotographs, we noticed that the periventricular layers, inner and outer SVZ, were quite different in the various lobes. Similarly, the fiber tracts also differed, and widely varied in thickness. Particularly in the frontal and parietal lobes, the intermediate zone (future white matter) was at this time point much wider than in the temporal lobe. In parallel, Tbr2+ and PCNA+ cells extended much farther into the IZ in FP areas than in the TC. In the TC, the anterior commissure seemed to represent an obstacle for the RGF, since it was traversed neither by the RGF nor did it contain Tbr2+ cells. We limited our study to the PSB and the adjacent regions, where the Tbr2+ progenitor cells became less and less numerous toward the IC (**Figures 8B,D**), and were basically absent at the level of the insula. The RGF was more difficult to discern than in earlier stages, but still very thick, prominent radial glia fibers emerged at the PSB and crossed the inner fibrous layer and adjacent IC (**Figures 8A,C**).

While at early fetal stages the PSB extended medially beyond the cortico-striatal sulcus, at midgestation it had shifted laterally in the temporal lobe, but not in the frontal lobe. Future studies will show how the PSB in different lobes behaves toward the end of cortical neurogenesis.

### Prenatal Development of Lamination in the Insula

**FIGURE 9:**
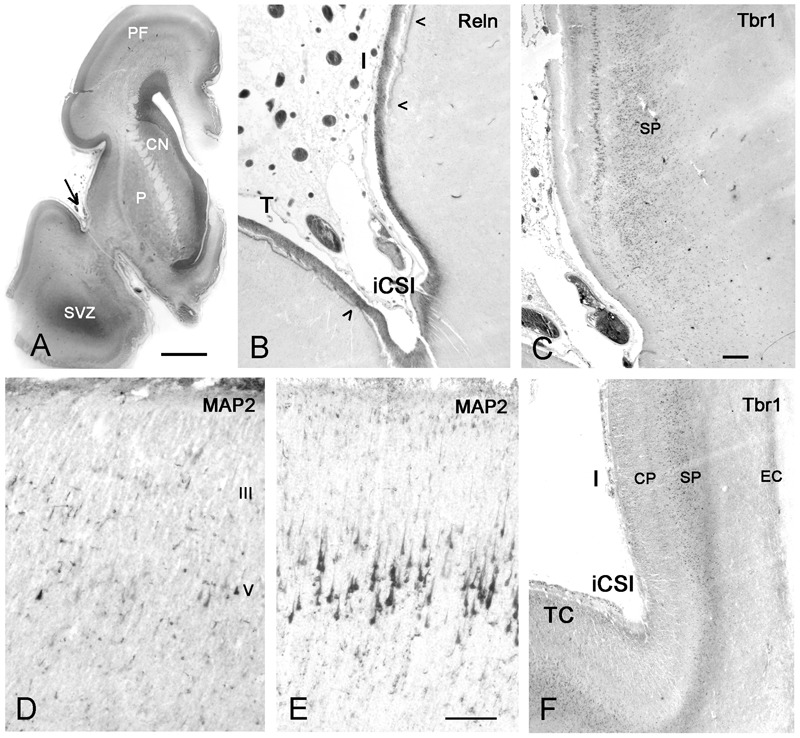
First appearance of lamination in the insula at midgestation (21GW). **(A)** The anatomy of a Nissl-stained hemisphere near the limen insulae. The arrow points to the inferior circular sulcus (iCSI) between the rostro-ventral insula and the temporal lobe. This region, shown in **B** (Reelin) and **C** (Tbr1), represents the transition between the ventral periallocortical insula and the dorsal isocortical insula. **(B)** The periallocortical insula lacks the Reelin+ plexus of the Cajal–Retzius cells in the lower marginal zone (arrowheads), characteristic of isocortex, although Cajal–Retzius cells are present all over the outer marginal zone. **(C)** A prominent layer V shows cytoplasmic Tbr1 staining, while the subplate has nuclear staining. At a more caudal level, MAP2 reveals a few pyramidal cells in layers V and III in the anterior insula **(D)**, which are much smaller and less numerous compared to the Betz cells in layer V of the primary motor cortex **(E)**. In **F**, Tbr1 marks the subplate (SP), which is continuous with the subplate of the adjacent temporal cortex (TC), although narrower and more compressed than the latter. CN, caudate nucleus; P, putamen; SVZ, SVZ of the temporal horn which opens at more caudal levels. Bars: in **A**: 400 μm; in **C**, for **B,C,F**: 160 μm; in **D**, for **D** and **E**: 75 μm.

During the first half of gestation, the insular cortex had a uniform structure, with a CP formed by densely aggregated immature neurons. On the whole, the insular CP was narrower than that of the adjacent opercular areas. The first clear appearance of layering was at midgestation (20/21 GW), when MAP2 (**Figure 9D**) and Tbr1 (**Figures 9C,F**) immunostaining (in maturing human cortical neurons, Tbr1 is cytoplasmic) indicated the presence of a distinct layer V, or inner pyramidal layer, which was particular prominent in the anterior agranular insula (**Figure 9C**). However, compared with the Betz cells in the adjacent primary motor cortex (**Figure 9E**), the dimensions and proportions of layer V pyramids in the anterior insula were rather reduced, as well as their positivity for MAP2. The deep layer VI and subplate were also Tbr1+, although in this case staining was nuclear (**Figure 9F**). The subplate was directly continuous with the subplate of the adjacent opercular cortices, but considerably reduced in width. Remarkably, the rostral and intermediate insula lacked an intermediate zone, characterized by horizontal fibers traversed by clusters of migrating neurons (Bystron et al., 2008; **Figures 6E,G, 8C**). In the posterior insula, the intermediate zone was present, traversed by calretinin+ fibers from the posterior limb of the IC (not shown).

Also at midgestation, we observed a difference in the distribution of the Reelin+ axonal plexus of the Cajal–Retzius cells in the lower marginal zone (Meyer and González-Gómez, 2017): The plexus, characteristic of isocortex and important for laminar arrangement of neurons, was absent in the periallocortical transition area (**Figure 9B**), but appeared in the more dorsal, isocortical insula. Cajal–Retzius cells were, however, abundant in the upper marginal zone all over the insula.

In the ages examined after midgestation, 24 and 25 GW, the overall immaturity of the insular CP persisted, and there was no evidence for a future differentiation into an agranular, dysgranular and granular cortex. Since at 32 GW the insula presented an adult-like morphology, cytoarchitectonic maturation would take place during the interval between 25 and 32 GW. Similarly, in our coronal sections at 24 and 25 GW, we were unable to distinguish incipient insular sulcation and gyration.

## Discussion

The insula derives from the PSB of all cortical areas adjacent to the Sylvian fissure: PF, frontal, parietal, and temporal, and thus constitutes a central node of the human cortex. Radial migrations from the PSB to the insula have to cross the growing IC, and circumvent the BG, and reach the insula via a RGF that course in the EC. According to its origins, the RGF has four main components, parts of which merge, and may determine the granular, dysgranular and granular character of the insular sectors.

### The Anatomy of the Developing Insula

The human insular lobe is shaped by the curvature of the lateral ventricle, and lies embedded between PF, frontal, and parietal areas dorsally, and temporal areas ventrally, from which it is separated by the Sylvian fissure and the circular sulcus. Its position external to putamen and IC prevents direct contacts with the periventricular proliferative zones, and radial migrations to the insula have to take indirect, unusual routes to reach and populate the distant lobe. Using radial glia architectonics and the pallial markers Tbr1 and Tbr2 (Hevner et al., 2003; Englund et al., 2005), we identified the PSB as the origin of the neurons destined to form the insular cortex. During early fetal development, the PSB follows the curvature of the ventricle, and thus extends from PF to temporal levels. We suggest that local differences of the PSB along its extent through the various lobes contribute to the multifaceted lamination pattern of the insular cortex. The different timing of the entrance of the IC into the cortex traversing the PSB (Krsnik et al., 2017), with rostral parts preceding more caudal parts, is an additional influencing factor, which will require further studies.

The finding that the architecture of the insula is contingent on size and orientation of the lateral ventricle and the curved shape of the PSB, explains the differences in insular structure reported in a variety of mammals. Even though comparative studies tend to emphasize common principles of brain structure in order to establish homologies (Cat: Clascá et al., 1997; monkey: Gallay et al., 2012; Evrard et al., 2014), anatomical studies in a wide variety of mammalian species usually not studied in the laboratory, including the dolphin (Jacobs et al., 1984; Casanova et al., 2010), demonstrated an extremely variable shape, general organization, lamination and cellular specialization of the insula, to the point that there is no recognizable common model of organization of the mammalian insular cortex (Butti and Hof, 2010). The relationship between insula and claustrum is similarly controversial. We neglected the claustrum, because we did not detect migratory mechanisms similar to those of the insula (Mufson and Mesulam, 1982; Nieuwenhuys, 2012). Developmental gene expression studies postulated that insula and insular claustrum are formed from the lateral pallium (Watson and Puelles, 2017). It may be argued that the mouse is not the best model for the human cortex, taking into account recent studies suggesting that the ancestor of mammals was probably a gyrencephalic animal (O’Leary et al., 2013; Lewitus et al., 2014). The magnitude of species differences suggests that the development of the insula is best understood when examined together with the anatomical landmarks that define this lobule in a given species.

In any case, the human insula should be considered in the context of its complex cognitive, social and emotional functions, including empathy, altruistic behavior, self-awareness, interoception, mindfulness, and consciousness (Craig, 2009; Fan et al., 2011; Fischer et al., 2016; Tusche et al., 2016; Laneri et al., 2017), or, as expressed by Craig (2010), the “sentient-self.” In keeping with these human-specific functions, a volumetric comparison of the insula of human and non-human primates revealed that in terms of absolute volumes the left and right agranular insula are among the most enlarged cortical areas relative to the chimpanzee (Bauernfeind et al., 2013). The degree of “granularity” of a cytoarchitectonic area is thus unrelated to its involvement in networks engaged in human cognitive functions such as those attributed to the anterior insula.

### The PSB Is the Origin of the Insula

The PSB has been extensively studied in terms of comparative anatomy, establishing homologies with the anterior dorsal ventricular ridge (ADVR) of sauropsids, and the fate of the cells in the LCS in sauropsids and mammals (Molnár and Butler, 2002), as well as in terms of developmental gene expression. Gene expression studies in the PSB of mice showed that the PSB is the main source of POC, claustrum, olfactory bulb, olfactory tubercle, and amygdala, and that the cells destined to these centers migrate ventrally via the LCS (Medina et al., 2004; Carney et al., 2006; Cocas et al., 2011). The insula as a PSB derivative has received less attention, probably because it is so unconspicuous in the rodent. The PSB is certainly an important landmark in the rodent brain, where it represents the boundary between pallium and subpallium, and is implicated in dorsoventral patterning of the telencephalon (Stoykova et al., 2000; Yun et al., 2001), but to what extent is it relevant for the human brain? Molnár and Butler (2002) recognized the evolutionary potential of the PSB, and also its function as an initial barrier zone for crossing cortico-thalamic and thalamo-cortical axons in the IC. However, the rodent studies did not take into account the enormous regression of olfactory structures in the microsmatic human, which rests importance from this aspect of the LCS. Instead, we show that the early descending migrations from the human PSB are mostly destined to the insula, although they also contribute Tbr1+ and calretinin+ cells to the POC. Furthermore, the rostro-caudal extension of the PSB is hugely increased due to the size increase of both, cortical progenitor zones and GE. Another important factor is the IC, which crosses the PSB (Molnár et al., 2012), and thus represents an additional obstacle for radial migration from the PSB to the insula. We suggest that the prominence of the PSB in human is an important factor for the development of the insular lobe. Furthermore, in the human brain, the insula does not represent the most lateral part of the cortex as in the rodent, but rather emerges as the core cortical region that makes possible the mirror arrangement of dorsal (FP) and ventral (temporal) cortical lobes. The central anatomical position of the insula in the human telencephalon is also paralleled by a similar central functional relevance, since especially the dorsal anterior insula can be considered a critical hub in connectivity networks of the human brain (Uddin et al., 2014).

### Radial Glia Architectonics Reveal a Migration Route from the PSB to the Insula

Radial glia has important roles in cortex development: It serves as a guidance substrate for radially migrating neurons (Rakic, 1971), and is also the principal progenitor cell type of the developing telencephalon (Malatesta et al., 2000; Miyata et al., 2001; Noctor et al., 2001; Tamamaki et al., 2001). In early embryonic stages, radial glia somata are confined to the VZ as apical radial glia, while in later stages, basal or oRG forms the proliferating cell population in the SVZ (Fietz et al., 2010; Hansen et al., 2010; Reillo et al., 2011). An oSVZ (Smart et al., 2002) is more prominent in gyrencephalic brains than in the lissencephalic rodent, and has been suggested to play key roles in the folding of the neocortex in gyrencephalic species because of its abundance in oRG that promote cortical expansion (Fietz et al., 2010; Hansen et al., 2010; Lewitus et al., 2013; Martínez-Martínez et al., 2016). On the other hand, comparisons of lissencephalic primates, gyrencephalic rodents, and carnivores suggested that the cytoarchitectonic subdivisions of the SVZ are an evolutionary trend and not a primate-specific feature, and that a substantial population of oRG exists unrelated to the degree of cortical folding (García-Moreno et al., 2012; Kelava et al., 2012; Martínez-Cerdeño et al., 2012, 2016). The expression of transcription factors Pax6 and Tbr2 varies between the various types of progenitor cells and species (Hansen et al., 2010; Kelava et al., 2012; Betizeau et al., 2013; Cunningham et al., 2013), although the expression of Tbr2 in neural precursor cells can be used for defining the boundaries of the SVZ both developmentally and evolutionarily (Martínez-Cerdeño et al., 2016). It is not clear whether also the intermediate progenitor cells, which lack a polarized process, express the pan-radial glia marker vimentin. In any case, the vimentin-expressing radial glia cells in the SVZ of our human material displayed the various morphotypes described in the monkey (Betizeau et al., 2013), where they indicate differential mitotic potentials and cell-cycle parameters. Radial glia-guided locomotion is the migration mode of excitatory cortical neurons on their route through intermediate zone, subplate and CP, until they reach the marginal zone, detach from the radial glia fiber, and change to a somatic translocation mode (Tissir and Goffinet, 2003; Sekine et al., 2011). The critical question is the degree of horizontal dispersion along the radial glia route. Radially migrating neurons may change from one radial glia fiber to an adjacent one, so that the strictly radial orientation of a radial glia fiber does not necessarily imply a similar radial course of the migrating neurons (Reillo et al., 2011; Gertz and Kriegstein, 2015). It is thus possible that the origin of neurons in the insula might be more extensive than described here, and that the SVZ of the adjacent opercula contribute neuroblasts dispersing tangentially into the insula. We suggest, however, that it is precisely the absence of tangential dispersion between adjacent lobes that leads to the enormous growth of the opercula, versus the restricted expansion of the insula. The distinct RGF into the insula is another argument for an origin from a specific sector of the oSVZ at the PSB. The RGF represents the main radial migration substrate from the PSB to the insula, and is set apart from the non-fasciculated oRG fibers originating from the oSVZ of the opercula. It is possible though that tangential dispersion within the spatially compressed RGF contributes to the inhomogeneities of layer IV. An important feature of the human RGF is that it is composed of descending and ascending fibers and thus connects the derivatives of the FP and temporal SVZ, which act in concert in the formation of the insula. It appeared, however, in our material that the dominant source of insular neurons is the dorsal PSB, whereas the temporal PSB contribution is less substantial. Future studies using region-specific markers might solve this question.

### The Variability of the Inner Granular Layer of the Human Insula

The human neocortex displays a large diversity of size and density of neurons, which are arranged in six horizontal layers of variable width. This diversity is the foundation of the cytoarchitectonic areas described by Brodmann (1909) and Von Economo and Koskinas (1925).

A major criterion in these classifications is the differentiation of the granular layers II and IV, which define the degree of granularity of a given area. Primary sensory areas have a particularly prominent layer IV, which is the main target of thalamo-cortical fibers, and populated by its principal neurons: spiny stellate cells, star pyramids, and small to very small “dwarf pyramids” (Von Economo and Koskinas, 1925). Spiny stellate cells are glutamatergic excitatory neurons (Conti et al., 1989; Feldmeyer et al., 1999), which establish asymmetric synapses mainly with dendritic spines (Saint Marie and Peters, 1985), give rise to interlaminar projections (Qi and Feldmeyer, 2016), and even connect adjacent areas (Meyer and Albus, 1981). In human auditory cortex, layer IV is populated by transitional forms between spiny stellate and small pyramidal cells (Meyer et al., 1989). However, layer IV is also prominent in many other cortical areas, such as the posterior insula, where anatomical and functional studies stressed its afferent sensory (gustatory, auditory, and vestibular) input (Augustine, 1996; Kurth et al., 2010).

There is a general agreement in the basic classification of the insula into agranular, dysgranular and granular parts (Mesulam and Mufson, 1985), even though the number of subareas varies between authors (reviewed by Nieuwenhuys, 2012). In our perinatal material we tried, following Von Economo and Koskinas (1925), to section the insula in a plane perpendicular to the main axis of most insular gyri. However, the insular gyri undergo subtle changes in orientation, have multiple dimples and small subsulci, which altogether distort the naturally vertical columnar arrangement of all layers including layer IV, and may give the impression of a distinct cytoarchitectonic subdivision.

As described here, the insula derives from the PSB, which in turn is continuous with the VZ and SVZ of the adjacent lobes. In this sense, the agranular character of the anterior insula reflects the trend of the agranular PF and frontal areas, whereas the granular caudal insula resembles the hypergranular character of the adjacent parietal and temporal sensory areas. The dysgranular character of the intermediate areas of the insula may be attributed to the contribution and possible intermixture of RGF compartments FP and T, as well as to the possibility of tangential dispersion within the RGF.

In the literature on the oSVZ, it is often emphasized that the subgranular layers V and VI derive from the VZ and inner SVZ, whereas the supragranular layers III and II originate from the oSVZ (e.g., Smart et al., 2002; Lukaszewicz et al., 2005; Nowakowski et al., 2016), leaving open the question of the origin of layer IV. Species differences may account for this apparent neglect. As shown by Martínez-Cerdeño et al. (2012), the peak in number of Tbr2+ progenitors and mitotic divisions in the oSVZ of the macaque somatosensory cortex occurs during the generation of layer IV, whereas in rat and ferret this peak is at the end of cortical neurogenesis, when the supragranular layers are born. It is thus tempting to propose that layer IV of the human insula derives predominantly from the oSVZ. The degree of granularity of a given cytoarchitectonic area may thus depend on the availability of oSVZ progenitors characterized by high-output cycling parameters during a precise time window (Betizeau et al., 2013), along with the presence of an adequate migration substrate. The generation of a cell-rich layer IV is an important issue particularly in the primate and human brain, where the classification into anatomical and cytoarchitectonic areas is largely based on the granularity of the inner granular layer. Interestingly, the “highest” cognitive functions within the insular lobe are attributed to the agranular anterior insula, which demonstrates that the agranular character of a cortical area is not an indication of less complex functions. It would be desirable that future ontogenetic and phylogenetic studies of the cerebral cortex become more focused on the peculiarities of the human brain (Clowry et al., 2010).

## Author Contributions

All authors listed have made a substantial, direct and intellectual contribution to the work, and approved it for publication.

## Conflict of Interest Statement

The authors declare that the research was conducted in the absence of any commercial or financial relationships that could be construed as a potential conflict of interest.
